# First adaptation of quinoa in the Bhutanese mountain agriculture systems

**DOI:** 10.1371/journal.pone.0219804

**Published:** 2020-01-16

**Authors:** Tirtha Bdr Katwal, Didier Bazile

**Affiliations:** 1 Agriculture Research and Development Center of Yusipang, Department of Agriculture, Ministry of Agriculture and Forests, Thimphu, Bhutan; 2 CIRAD, UPR GREEN, Montpellier, France; 3 GREEN, Univ. Montpellier, CIRAD, Montpellier, France; 4 CIRAD, DGDRS, Montpellier, France; Potsdam Institute for Climate Impact Research, GERMANY

## Abstract

Bhutan represents typical mountain agriculture farming systems with unique challenges. The agriculture production systems under environmental constraints are typical of small-scale agricultural subsistence systems related to family farming in the Himalayan Mountains with very low level of mechanization, numerous abiotic stresses influenced by climate and other socio-economic constraints. Quinoa was first introduced in 2015 through FAO’s support to Bhutan as a new crop to enhance the food and nutritional security of the Bhutanese people. The main objective was to adapt this versatile crop to the local mountain agriculture conditions as a climate resilient crop for diversifying the farmer’s traditional potato and maize based cropping systems. Ten quinoa varieties were evaluated at two different sites representing contrasted mountain agroecologies in Bhutan and were tested during the two agricultural campaigns 2016 and 2017. Yusipang (2600 masl) represents the cool temperate agroecological zone, and Lingmethang (640 masl) the dry subtropical agroecological zone. The sowing time differed depending on the growing season and elevation of the sites. Results indicate that quinoa can be successfully grown in Bhutan for the two different agroecological zones. The grain yields varied from 0.61 to 2.68 t.ha^-1^ in the high altitude areas where quinoa was seeded in spring and harvested in autumn season. The grain yield in the lower elevation ranged from 1.59 to 2.98 t.ha^-1^ where the crop was sown in autumn and harvested in winter season. Depending on genotypes’ characteristics and agroecological zones, crop maturity significantly varied from 92 to 197 days with all genotypes maturing much earlier in the lower elevations where mean minimum and maximum temperatures during the growing season were higher. Quinoa is rapidly promoted across different agroecological contexts in the country as a new climate resilient and nutrient dense pseudo cereal to diversify the traditional existing cropping system with some necessary adjustments in sowing time, suitable varieties and crop management practices. To fast track the rapid promotion of this new crop in Bhutan, four varieties have been released in 2018. In just over three years, the cultivation of quinoa as a new cereal has been demonstrated and partially adapted to the maize and potato based traditional cropping systems under the Himalayan mountain agriculture. Quinoa is also being adapted to the rice based cropping system and rapidly promoted as an alternative food security crop in the current 12^th^ Five Year national development plan of Bhutan. To rapidly promote quinoa cultivation, the Royal Government of Bhutan is supporting the supply of free quinoa seeds, cultivation technologies and milling machines to the rural communities. To promote the consumption and utilization of quinoa at national level, consumer awareness are created by preparing and serving local Bhutanese dishes from quinoa during local food fairs and farmer’s field days. In addition, the Royal Government of Bhutan has included quinoa in the school feeding programme recognizing the high nutrient value of the crop for enhancing and securing the nutritional needs of the young children.

## Introduction

Bhutan is a small land locked country with a fragile mountainous environment located in the Himalayan foothills. It is located between China and India with a geographical area of 38,394 square kilometers and a total population of 735,553 persons [1]. It represents a Least Developed Country with a per capita GDP of US$ 2879.07 [2]. “The Least Developed Countries (LDCs) is a list of developing countries that exhibit the lowest indicators of socioeconomic development, with the lowest Human Development Index ratings of all countries in the world” according to the United Nations. Bhutan is mostly mountainous with over 70% of its area under forest. The country is primarily dependent on agriculture and about 62.2% of the population is engaged in subsistence agriculture for their livelihood [3]. Despite agriculture being the primary sector, the country is only able to secure 60% of its cereals, vegetables and animal product needs through domestic production and has to buy over 40% of its food requirement through imports [3]. Bhutanese agriculture represents typical subsistence mountain agriculture where smallholder farmers follow an integrated family farming where agriculture, livestock and forests are intricately linked to meet the household food security. Majority of the farmers grow crops, rear livestock for food, manure and draught power and depend on forest for fuel, fodder, food, litter and timber. To enhance domestic food production and diversify the farmers existing cropping system, quinoa, a new crop was introduced for the first time to Bhutan in 2015 from Peru [4–5] with the support of the Food and Agriculture Organization (FAO) of the United Nations. The rationale for the introduction of quinoa to Bhutan is that FAO has identified quinoa as the most potential crop that can offer food and nutrition security to the world in the next century [6–8].

Lambsquarters (*Chenopodium album*), a quinoa crop wild relative originated from Eurasia, was domesticated in the Himalayan Mountains and it is still possible to find farmers who grow it in India, Nepal, China and Bhutan for its grain or leaves [9–10]. It is a historical fact that the use of Chenopodium leaves and seeds for human consumption is not exclusive to the Andean region. A species of Chenopodiaceae was cultivated in the Himalayas a long time ago at altitudes of 1 500–3 000 masl [11–12]. This species was classified as *Chenopodium album* but also as an unidentified Chenopodium sp., belonged to a complex of two species *C*. *album* and *C*. *quinoa* and grown in the Himalayan regions of Punjab. In Bhutan, some farmers continue to grow it and some Chenopodium sp. as food and vegetables but the identification of species is until today not clear.

In addition, since the eighties, a researcher is growing quinoa on the Tibetan plateau, which presents cold high-desert farming conditions similar to those found on the Altiplano in the Andes. The initial objective was to diversify the mainly barley-based diet of Tibetans by adding vegetable proteins to it through quinoa. The first project was launched in 1984 and the first quinoa plantings took place in 1988. After undergoing training in plant breeding in Mexico and Hawaii, Dr Trashi Gongbu succeeded in adapting quinoa to this mountain region and has now bred local varieties. The area under cultivation is still limited to a few hundred hectares mainly due to difficulties of accessibility. Nevertheless, efforts for adaptation and ongoing work with small farmers have raised yields to nearly two tons per hectare. The painstaking work of plant breeding over different periods through crossing of genetic material from southern Chile, Bolivia and quinoa varieties developed for Mexico has created a new biodiversity of quinoa for high-altitude Himalayan contexts [13–14].

More recently quinoa trials were conducted in the northern India plains which is quite close to the Himalayan region and have reported that the potential of expanding quinoa cultivation in the Himalayan region is very high [15–16]. Nowadays, several studies [5–6] have established that quinoa can be cultivated in different growing environment with humidity range of 40 to 90%, at altitudes varying from sea level to 4500 masl and has the ability to tolerate temperature variation from -8°C to 38°C [17–18].

The fundamental objectives of introducing quinoa is to diversify the farmer’s traditional cropping systems through adapting this versatile crop to the Bhutanese subsistence mountain farming systems as a climate resilient crop in order to enhance the food and nutritional security of the Bhutanese people [4]. This paper highlights the results of quinoa demonstration undertaken in 2015, and presents the detail results of replicated trials conducted at two locations in 2016 and 2017.

## Materials and methods

### Bhutanese’s mountain agriculture environments

Before describing the target-growing environment for quinoa adaptation in Bhutan, it is important to provide an overview of the situation of the Bhutanese mountain agriculture where the new quinoa crop is expected to adapt and fit into the existing traditional farming systems. By virtue of being located in the Himalayas, Bhutan is mostly dominated by rugged and steep topography. There is a very large altitudinal variation starting from 100 meters in the south and rising to more than 7,000 masl in the north. The farming environment is physically challenging with a mountainous terrain. About 5.7% of the total geographical area has a gradient above 100% (45° angle), which is prone to very severe soil erosion and soil stability. 43.8% of the area has a slope ranging between 50–100% and 36.5% of the area has slope between 25–50%, while only 14% of geographical area has slope of 0–25% [19], which indicates the adverse nature of the agriculture land topography. Bhutan is divided into three distinct climatic zones, which are alpine, temperate and subtropical zones. Equally unique is the agroecological zones, which are subcategorized into six major groups corresponding with altitude and climatic conditions for agriculture planning and coordination (Table 1). The alpine and the cool temperate zone, which is dominated by mountainous terrain, has about 53% of the geographical area, which clearly indicates the geo-physical setting of the country. Such a fragile geo-physical setting dominated by steep topography makes Bhutan highly vulnerable to any small variation in the weather patterns and the current effects of climate change.

**Table 1:** Major agroecological zones of Bhutan.

Agroecological zones	Altitude (m.asl)	Temperature (^ₒ^C)		Mean Rainfall (mm)	Proportion of Geographical Area (%)
		Max	Min		
Alpine	3500–7500	12.0	-1.0	<650	28.6
Cool temperate	2600–3600	22.0	1.0	650–850	23.9
Warm Temperate	1800–2600	26.0	1.0	650–850	18.6
Dry Sub-tropical	1200–1800	29.0	3.0	850–1200	13.1
Humid Sub-tropical	600–1200	33.0	5.0	1200–1500	10.2
Wet Sub-Tropical	100–600	35.0	12.0	2500–5500	5.6

Adapted from Renewable Natural Resources Research Strategy and Plan Document 1992, Ministry of Agriculture, Planning and Policy Division, Thimphu, Bhutan

The cultivated agriculture area is estimated to be only 2.93% of the national geographical area [20]. There are three dominant agriculture land use categories: i- *Chhuzing* (or Wetland) which are terraced paddies for rice cultivation; ii- *Kamzhing* (or dryland) which are rainfed lands that are not terraced and bunded; and iii- Horticulture land under orchards and plantations. The predominant crops under orchard and plantations are citrus, apple, arecanut and large cardamom. Among these three land use categories *Kamzhing* is most dominant and constitutes 61.90% of the agriculture area, *Chhuzhing* covers 27.86% and 10.24% is under orchards and plantations [20]. There are three main distinct cropping systems, which include rice, maize and potato based systems with different forms of multiple cropping as one of the simple mechanisms to produce more per unit area and to limit risks [21].

The dominant cropping systems and crop rotations for different agroecological zones are briefly summarized in Table 2. In the *Kamzhing* under the cool and warm temperate zones is potato, wheat or apples based where other crops such as vegetables, mustard, and buckwheat are rotated with cereals or intercropped in orchards. In the dry and humid subtropical areas, maize based cropping systems are predominant where other cereals such as millets and buckwheat, vegetables, legumes and oilseeds are cultivated. Maize + potato intercropping and various forms of multiple cropping are predominant under *Khamzing*. In the terraced wetland or *Chhuzhing* under in the warm temperate zone, farmers mostly grow a single crop of high altitude irrigated rice with some farmers rotating peas, potato, oat and wheat as fodder after rice. The cultivation of a second crop after rice is limited by incidence of early frost and short growing season. In the *Chhuzhing* under wet and humid subtropical areas, rice is followed by mustard, wheat and vegetables in small areas as water is limiting factor after the rice season.

**Table 2:** Dominant agriculture land use categories, crops and cropping sequences.

Agroecological zones	Altitude (*masl*)	Land Use Types, Major Crops and Cropping Systems		
		*Khamzing*	*Chhuzing*	Horticulture/Plantations
Alpine	3500–7500	Pasture	Absent	Absent
Cool temperate	2600–3600	Barley- fallow / Potato—Turnip	Absent	Apple
Warm temperate	1800–2600	Potato- Buckwheat / Potato-Turnip / Wheat/Barley- Buckwheat / Potato- Wheat/Barley, Vegetables- Wheat	Rice-Fallow / Rice-Potato / Rice-Peas / Rice- Wheat	Apple, Walnut, Pear, Peach, Plum
Dry sub-tropical	1200–1800	Maize+ Potato / Maize+ Soybeans / Maize- Mustard / Maize- Barley / Maize- Fodder Oat / Maize- Buckwheat / Chili- fallow, Vegetables- Wheat	Rice- Wheat / Rice- Mustard / Rice- Chilli / Rice- Vegetables	Apples, Pears, Peach, Kiwi, Large Cardamom
Humid sub-tropical	600–1200	Maize- Maize / Maize- Broad Beans *(Rajma) /* Maize- Millet / Millet- Fallow / Maize–Buckwheat / Maize- Potato / Maize- Buckwheat Vegetables-Pole Beans- Dwarf beans / Maize + Finger Millet / Buckwheat-Millets	Rice-Fallow / Rice-Mustard / Rice Wheat / Rice-Buckwheat / Rice- Vegetables / Rice-Chilli	Citrus, Large Cardamom, Mango, Avocado, Banana
Wet sub-tropical	100–600	Maize- Mustard / Maize-Maize / Maize- Grain Legumes (Back gram, rice bean, broad beans) / Maize + Millet / Foxtail Millet- Finger Millet	Rice- Fallow / Rice-Maize / Rice-Wheat / Rice-Sesbania / Rice-Buckwheat	Arecanut, Mango, Avocado, Banana, Litchi

Adapted and updated from Katwal previous works [21]

Farm mechanization is highly limited due to steep landscape and as a result, the cost of production for the production of different commodities is generally very high. Of the 112,550 hectares (ha) of cultivated area only 32,718 ha is irrigated which means only 29% of the agriculture area is under irrigation [19]. Thus, crop production predominantly depends on seasonal monsoon rains that normally start from late June to September [22] and water for crop production is increasingly becoming scarce and unpredictable due to the increasing variation of precipitation pattern.

Majority of the Bhutanese farmers practices self-sustaining, integrated and subsistence agricultural production systems with an average land holding of about three acres (1.215 ha) where farmers grow a variety of crops under different farming practices and rear livestock to meet their household food security. Owing to the topography dominated by high mountains and deep valleys, there is a wide variation of microclimate, which requires highly location specific crops and varieties. Many of these crops, varieties and livestock breeds, are best adapted to marginal mountain farming environments for which finding suitable replacements are not straightforward [23].

The average landholding per household is 3.0 acres, which is further decreasing due to the rapid rate of land fragmentation that is brought about by the practice of land distribution among children as a right to family land inheritance. Bhutan’s agriculture system is largely traditional with a very minimal use of external inputs like inorganic fertilizers and pesticides. The use of external inputs like chemical fertilizers and plant protection chemicals for agriculture production is estimated to be used by 37% of the farmers in about 19% of the cultivable land, which implies 162,000 acres of cropped area is chemical free [24] making it principally organic by default. Most of the cultivated soils are estimated to have high organic matter content and some soils even exceed 6% organic matter [25]. The National Soil Service Centre estimates that the inorganic fertilizer use in 2016 was 11.9 kg.acre^-1^, which is low compared to 13.66 kg.acre^-1^ in 2015. In the traditional farming, the application of Farm Yard Manure (FYM) continues to be the major source of plant nutrients, which is applied at the rate of 3 to 5 t.ha^-1^ [25].

The country has four distinct seasons namely spring, summer, autumn and winter. The spring season, which is generally dry, starts in early March and lasts until mid-April. The summer season commences in mid-April with occasional showers and continues through the early monsoon rains of late June. The summer monsoon starts from late June through late September that brings heavy rains from the southwest [22]. The monsoon brings heavy rains, high humidity, flash floods and landslides, and numerous misty overcast days. The autumn season starts from late September or early October to late November. The dry and cold winter season commences from late November until March. During the winter months occurrence of frost in most part of the country is common and frequent snowfall is experienced at elevation above elevations 3,000 meters.

### Production challenges of mountain agriculture

The most pressing farming constraints of the subsistence for Bhutanese farmers are small land holding, dependency on monsoon rains, low farm productivity, high cost of production, low scope of farm mechanization owing to a mountainous terrain and, low volume of production and distance from the market. Because of the steep topography, the scope of farm mechanization is highly limited which makes that all agriculture operations are labour intensive. The most pressing abiotic challenges are varying precipitation patterns, a short growing season due to the early initiation of frost in the warm temperate and cool temperate areas and increasing climate extremes like drought, hail and windstorms, flash floods and increasing infestation by pests and diseases. Other climate induced abiotic stresses like drought, high temperature, and cold temperature and frost damages to crops decrease farmer’s choices of crops and ability to increasing cropping intensity. A unique and the most pressing problem confronting subsistence Bhutanese farmers is the increasing human wildlife conflicts where crop damage and livestock killings by wildlife are forcing farmers to give up farming. According to the State of Environment Report [22], 55% of the crop damage is attributed to wildlife mainly elephants, wild pigs, deer, monkeys, porcupine and different species of birds. Bhutan has a very strong environment conservation policy, which is also one of the four pillars of Gross National Happiness philosophy. The constitution of the Kingdom of Bhutan requires maintaining a minimum of 60% of the area under forest cover. The large proportion of national land area is under forest cover and 51.40% of the country’s landscape under protected area system that serves as the key habitat for wildlife often attributed for the increasing wildlife impact on agriculture.

Climate change and its impact on subsistence Bhutanese agriculture is an emerging issue where the need for climate resilient crops and climate smart technologies is a priority. To address the increasing impacts of climate-induced stresses there is a need to identify and use stress tolerant species, which exists but are neglected and underutilized [8, 26, 27]. It has been reported that quinoa has a high degree of resistance to frost and, is known to survive a -8°C up to 4 hours depending on the crop stage and variety [28–30]. Bhutanese mountain agriculture currently faces several hostile production challenges of which most are related to weather and climate. Quinoa has been proven to have exceptional tolerance to hostile environments and is considered a good candidate crop that can help enhance food and nutritional security in the face emerging challenges imposed by climate change [8]. Quinoa is proven for its extreme agroecological adaptability and can solve crop adaptation problems in places where climatic and soil conditions are main limiting factors for crop production [31]. From its first introduction in 2015, quinoa has been included as a priority crop in the current 12^th^ Five Year Plan of the DoA [32–33]. Now, quinoa is rapidly being evaluated as a climate resilient crop under two different and contrasted agroecological zones with very specific microenvironments.

### Trials’ design

**Fig 1:**
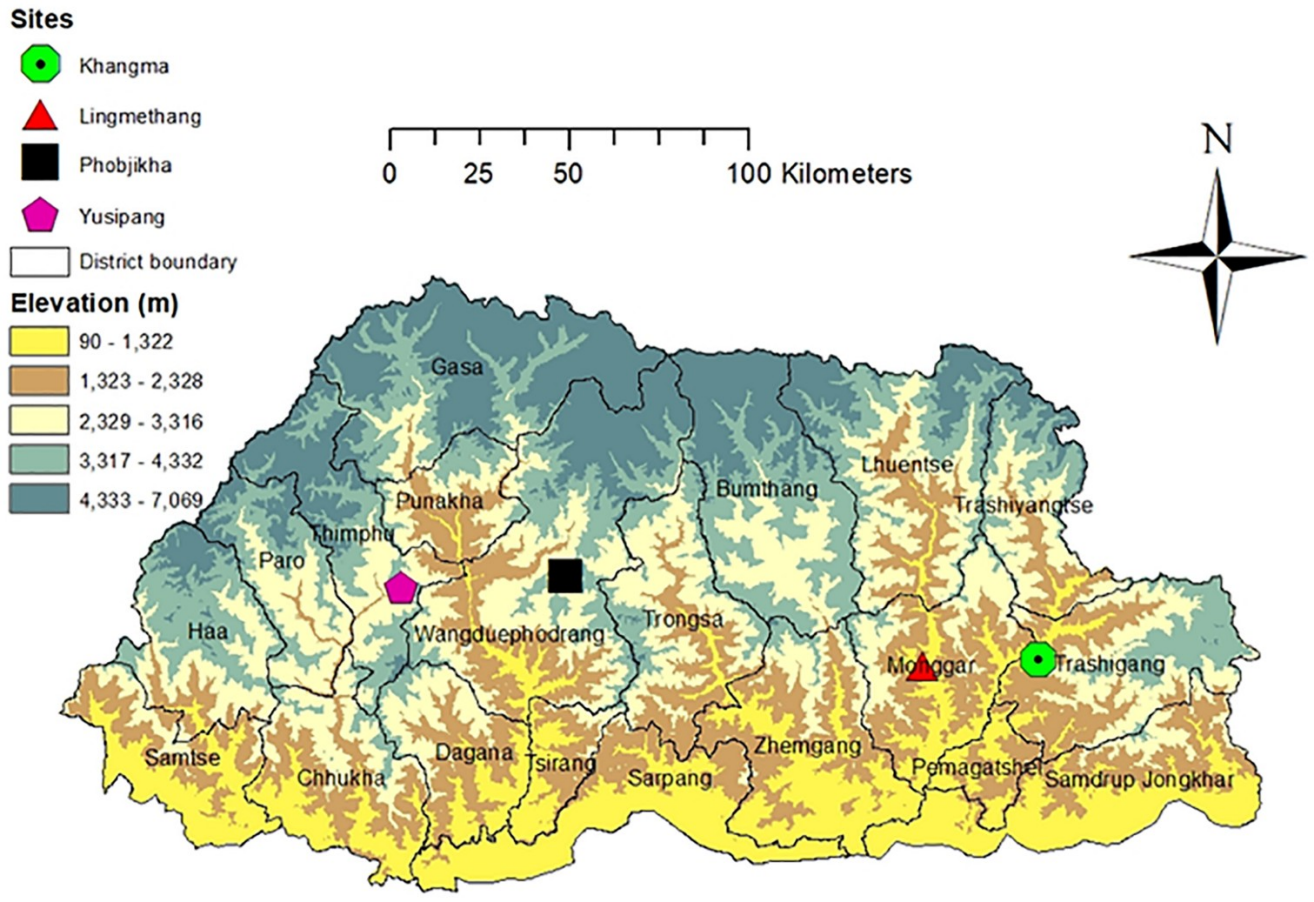
Elevation map of Bhutan with the location of the two study sites. Elaborated by the 1^st^ author TK at Ministry of Agriculture, Bhutan.

First demonstrations of quinoa in Bhutan were conducted in 2015 at Yusipang (2600 masl), Phobjikha (2900 masl) and Khangma (2100 masl) in the research farms with two varieties namely Amarilla Marangani and Amarilla Sacaca (or INIA 427) originated from Peru as quinoa commercial seeds [34]. The demonstrations were successful and produced good yields ranging from 2.31 to 2.24 t.ha^-1^ [4]. In 2016, eight more varieties (Blanca de Junín, Salcedo INIA, Altiplano, INIA 415 Pasankalla, INIA 420 Negra collana, Hualhuas, Huancayo & INIA 433 Santa Ana) were received from FAO-Peru (Project TCP/RAS/3411 coordinated by FAO-RAP) and, evaluated in six different locations [4]. Apart from these trials and demonstration, two separate quinoa adaptation trials were conducted at Agriculture Research and Development Center (ARDC) research farm at ***Yusipang*** under Thimphu Dzongkhag and at Agriculture Research and Development Sub-Center (ARDC SC) at ***Lingmethang*** under Mongar Dzongkhag (Fig 1). Dzongkhag signifies an administrative district in Bhutanese language. In both locations, the trials were conducted for two consecutive years in 2016 and 2017. In parallel, a joint study was conducted by the International Center for Tropical Agriculture (CIAT) and the Ministry of Agriculture and Forestry (MoAF) for undertaken to assess the impacts of climate change on five key crops (i.e. rice, maize, potato, chili and tomato) and three diversification crops (i.e. quinoa, kiwi and cardamom) [35]. The agronomic trials were repeated for two years because quinoa is a new crop to Bhutan and the variation in its performance needed to be precisely confirmed for confidently promoting this new crop in the farmers’ field. The different quinoa genotypes introduced to Bhutan were evaluated under rainfed dryland represented by both the two trial sites. A total of 10 varieties were evaluated at Yusipang, while at Lingmethang only nine varieties (without DoA-1- PMB-2015) were tested in both years. The ten varieties were the eight received from Peru in 2016 and two others namely Ivory 123 accessed from India and DoA-1-PMB- 2015 shared by one individual. The trial design used was a Randomized Complete Blocks (RCB) with three replications with a plot size of 10 m^2^. At Yusipang, there were 30 plots whereas at Lingmethang the number of trial plots used was 27. Each plot had four rows with a row-to-row spacing of 0.50 m and the plant-to-plant spacing was maintained at 0.25 m by thinning. The Indian fertilizers called *Suphala* (16:16:16 NPK) was applied at the rate of 70 kg.ha^-1^ and was uniformly incorporated in the furrows drawn with spade before seeding. The seeds were sown uniformly in line and covered with a thin layer of soil using a locally made broom. Weeds were controlled by three hand weeding. The trial was irrigated twice using micro sprinklers before flowering when the crop showed symptoms of moisture stress.

Field preparations were done mechanically using powertiller. To prepare the necessary fine seedbed for quinoa, two ploughing were done followed by the pulverization of soil with a rotavator to prepare it for good sowing. In both locations about 3 Mt of Farm Yard Manure (FYM) were applied and incorporated in the soil during field preparation. The seed plots were leveled with rakes and 2–3 cm deep furrows were marked uniformly with spade at a spacing 0.50 m. The seeds were uniformly broadcasted in the furrows apparently form lines and covered with a thin layer of soil using a locally made broom.

In both Yusipang and Lingmethang sites, when the leaves started turning pale yellow, leaf senescence was observed, and grains in the inflorescence became hard on pressing with fingers so the crop was considered ready for harvest. The whole plot was harvested manually and the samples from the trials were tied into bundles and dried by hanging in the shade for 10–15 days for curing. The physiological maturity of grains was determined when the seeds from the main panicle were resistant to crushing when pressed between two fingers. The samples were manually threshed and cleaned using local winnowers to obtain the grains for yield estimation. The data from the trials were entered in Microsoft Excel and analyzed with SPSS software. An ANOVA (analysis of variance) was used to compare the means with level of significance set at 5% (p ≤ 0.05). When performing a hypothesis test in statistics, the p-value helps for determining the significance of the results. As the p-value is a number between 0 and 1, it is interpreted in the following way where a small p-value (typically significance if p ≤ 0.05) indicates strong evidence against the null hypothesis, so you reject the null hypothesis. For analyzing our data, the results also present the standard error (SE) of the statistics as the standard deviation of the sampling distribution.

#### Description of the trial sites

At Yusipang, the trial was conducted in the research farm at an altitude of 2600 masl. Yusipang is located at latitude 27° 27.5’ to 27° 28.2’ N and longitude 89° 42.5’ to 89° 42.8’ E. The site represents a cool temperate agroecological zone and a typical highland mountain farming environment where crop cultivation is limited by cold temperature and frost. The incidence of frost starts by the second fortnight of October and continues till April. Farmers mostly cultivate temperate fruits like apples, peach, plum, walnut and pears. The predominant annual crops cultivated are potato and vegetables mainly the Cole crops. Being a peri-urban area there is a very good opportunity to market any agriculture products and hence the drive to grow fresh vegetables and other agriculture crops is very high.

The predominant soil texture of the site is sandy loams and sandy clay loams [36]. The soils at Yusipang research farm are very acidic to slightly acid, with pH (water) values in the range 4.8 to 6.5. Organic matter levels are low to moderate, with organic carbon contents of about 2.5% and C:N ratios are in the range 10 to 20. Available phosphorous levels are variable but are mostly moderate to high [36]. The mean minimum temperature ranges from -0.9°C in January to 15.4°C in July while the mean maximum temperature varies from 4.5 °C in January to 19.1°C in August. Mean annual rainfall was 860 mm from 2007 to 2017 (Table 3).

**Table 3:** Mean temperature and precipitation of two trial sites from 2007 to 2017.

Months	Jan	Feb	Mar	Apr	May	Jun	Jul	Aug	Sep	Oct	Nov	Dec
Yusipang* **(2600 *masl*)**												
Mean Minimum Temperature °C	-0.9	2.1	4.4	7.5	9.8	14.2	15.4	15.3	12.5	7.3	4.7	1.3
Mean Maximum Temperature °C	4.5	8.3	11.7	13.6	16.7	18.7	19.0	19.1	17.8	15.2	9.1	7.5
Mean Rainfall (mm)	20.4	11.7	10.7	30.6	31.4	174.2	192.6	164.4	129.6	77.4	1.2	16.0
Lingmethang** **(640 *masl***)												
Mean Minimum Temperature °C	9.4	11.1	15.4	18.6	21.2	23.7	24.2	23.7	22.5	18.6	13.1	11.9
Mean Maximum Temperature °C	23.5	23.3	26.9	30.1	32.3	32.8	33.4	32.8	32.4	30.8	27.8	26.2
Mean Rainfall (mm)	8.1	23.3	23.3	57.1	103.5	143.1	138.4	134.6	104.2	46.0	9.1	1.1

*Data Source for Yusipang: Forestry Research Program, UWICER, Yusipang and RNRRC Annual Reports

** Data Source for Lingmethang: National Center Hydrology and Meteorology, Ministry of Economic Affairs

At Yusipang, the trial was established on 18^th^ March in 2016 and on 23^rd^ March in 2017. Harvesting was done depending on the maturity of the varieties which started in September and was completed by end October for all the 10 varieties.

At Lingmethang, the study site is situated at 27° 15’ 42” N and between 91° 10’ 38” and 91° 11’ 17” E at an altitude of 640 masl. This quinoa trial was conducted at the research farm of the Agriculture Research and Development Sub-Center, which is administratively under ARDC Wengkhar. It represents a dry-subtropical agroecological zone. The main limiting factors for crop production in the dry-subtropical zone are varying precipitation pattern: rainfed farming that depends on monsoon rains, steep terrain with land slope of 25–50%, and high temperature in summer months especially in valley bottoms. Maize and potato intercropping is very popular. Farmers also cultivate different vegetables, grain legumes, millet, buckwheat, barley and mustard in the dryland.

The soils at Lingmethang are slightly gravelly to extremely gravely and slightly acid to neutral with pH values (water) all above 5.5. The organic carbon and total nitrogen are generally low to moderate and the overall fertility potential and inherent fertility is categorised as slightly poor [37]. The mean minimum temperature ranges from -0.9°C in January to 15.4°C in July while the mean maximum temperature varies from 23.4°C in January to 33.4°C in July. The mean annual rainfall was 792 mm from 2007 to 2017 (Table 3).

In 2016, the trial at Lingmethang was planted on 20^th^ October and the harvesting dates started in 2017 from 1^st^ February to 7^th^ March. The same was trial repeated in 2017 for which the sowing was done on 15^th^ September and the harvesting started from 20^th^ December 2017 and was completed by 7^th^ March 2018.

## Results

### Climatic conditions for quinoa adaptation

The climate, cropping season and farming practices of the two trial sites represent a typical mountain environment. The temperatures and precipitation are much lower during the winter season and the temperature and precipitation during the summer months are higher (Table 3). The winters are much severe in the warm temperate and cool temperate agroecological zones (> 1800 masl) where frost, low precipitation and short growing period is one of the major factors limiting the crop production and choice of crops. In the dry, humid and wet sub-tropical agroecological zones, the maximum summer temperatures can go above 30°C during April to September, which affects seed setting, and higher precipitation causes vivipary in quinoa. Considering the temperature and precipitation that determine the growing season, quinoa was sown from March to April in the warm and cool temperate agroecological zones and from September to November in the dry, humid and wet sub-tropical agroecological zones. In areas above 1800 masl represented by Yusipang, quinoa sown from end March to mid-April was successful with appreciable yields. The crop sown from September in areas less than 1800 masl, represented by Lingmethang, produced good yield and grain quality as the harvest season coincided with dry weather. The soil conditions of both the trials sites were suitable for quinoa with pH values that varied from 4.8 to 6.5 It has been reported that quinoa can tolerate wide range of soil pH from 4.5 to 9 [38].

### Yusipang study site

At Yusipang (2600 masl), the statistical analysis of crop maturity (p<0.001) and yield (p<0.003) showed significant differences among the ten varieties evaluated but there was no difference (at p ≤ 0.05) in plant height which is a first indication of biomass yield (Table 4). Under the cool temperate agroecology (2600–3600 masl) represented by Yusipang, the days to maturity of the 10 varieties ranged from 100 to 197 days (Table 4) with most of them in the group d > 178 days. The DoA-1-PMB-2015 was the earliest variety, which matured after only 100 days from sowing date. Based on the days to maturity, the 10 varieties can be classified into early and late maturing groups. Three varieties maturing in less than 150 days are early while those taking more than150 days are considerate late (Table 4). The yield of the 10 varieties ranged from 0.61 to 2.68 t.ha^-1^ (Table 4). The highest yield of 2.68 t.ha^-1^ was recorded for Ivory 123. However, no significant difference was found to the maturity and correlated to the precocity of the varieties.

**Table 4:** Mean plant height, maturity and yield of quinoa varieties at Yusipang (means of 2016 and 2017).

Variety	Seed Source	Plant Height(cm)	Maturity (days)*	Yield (t ha^-1^)
DoA-1- PMB-2015	No Known	103.83	100^a^	1.55^ab^
Ivory 123	India	113.68	128^ab^	2.68^b^
Salcedo INIA	Peru	131.68	145^bc^	0.88^a^
INIA 420 Negra Collana	Peru	107.58	178^cd^	0.90^a^
INIA 415 Pasankalla	Peru	121.28	183^cd^	1.13^a^
Amarilla Marangani	Peru	147.13	197^d^	1.32^ab^
Blanca De Junin	Peru	137.95	197^d^	0.61^a^
Hualhaus	Peru	144.42	197^d^	1.48^ab^
Huancayo	Peru	138.58	197^d^	0.79^a^
INIA 427 Amarilla Saccaca	Peru	155.05	197^d^	1.68^ab^
p		ns	<0.001	<0.003
SE ±		17.56	11.72	0.44

*means followed by same letters are not significantly different

### Lingmethang study site

At Lingmethang (640 masl), the statistical analysis of crop maturity (p<0.001) and yield (p<0.01) showed significant differences among the nine varieties tested but there was no difference (at p ≤ 0.05) in plant height (Table 5). The duration of crop maturity was much shorter at Lingmethang and ranged from 92 to 119 days after sowing date (Table 5). The short crop maturity can be explained by the higher mean minimum and maximum temperature (9.4 to 23.5°C) during the growing season from September to February as compared to Yusipang (Table 3). The yield of the nine varieties ranged from 1.59 to 2.98 t.ha^-1^ (Table 5), which the lower yield was much higher as compared to the lower yield of Yusipang (0.61 t.ha^-1^). The highest yield of 2.98 t ha^-1^ was also recorded for Ivory 123.

**Table 5:** Mean plant height, maturity and yield of quinoa varieties at Lingmethang (mean of 2016 and 2017).

Variety	Seed Source	Plant Height(cm)	Maturity (days)*	Yield (t ha^-1^)
INIA 415 Pasankalla	Peru	106.00	92^a^	1.59^a^
Ivory 123	India	126.50	100^ab^	2.98^b^
INIA 427 Amarilla Saccaca	Peru	139.67	102^abc^	2.37^ab^
Salcedo INIA	Peru	115.67	104^abc^	1.79^ab^
INIA 420 Negra Collana	Peru	118.53	107^abc^	1.69^a^
Amarilla Marangani	Peru	140.33	114^bc^	2.38^ab^
Huancayo	Peru	134.00	114^bc^	2.51^ab^
Blanca De Junin	Peru	145.57	119^c^	1.95^ab^
Hualhaus	Peru	143.57	119^c^	2.44^ab^
p		ns	<0.001	<0.01
SE ±		14.89	5.29	0.37

*means followed by same letters are not significantly different

Quinoa was sown in September as a second crop after maize. The mean maximum temperature at Lingmethang during May to August ranges from 30.1 to 32.4 °C (Table 3) which could affect grain setting. Further, if the crop is sown in February and March, the crop will be exposed to moisture stress in the early growth stages and harvesting will fall during peak monsoon that affects drying, curing and grain quality.

## Discussions

The results from the two study sites representing two types of agroecologies with different growing environments show that crop maturity and yield vary quite significantly both within and between varieties. The time taken for maturity of all the varieties was much shorter at Lingmethang (92–100 days) as compared to Yusipang (100–197 days), due to the effects of altitude on temperature and affecting the plant development during the agricultural season. The data on crop maturity, which is a measure of growing period from seed sowing to harvest from both the sites, confirms to the findings that different quinoa genotypes have showed different growing periods [38]. Jacobsen [39] has reported that under European conditions the growing periods for different quinoa genotypes varied from 109 to 182 days where as in South America the growing period recorded for different genotypes ranged from 110–190 days [40]. According to Jacobsen’s research [7], an ideal quinoa variety for grain production should have uniform maturity and early with a growing period of less than 150 days under European northern conditions.

At Lingmethang, the total grain yield obtained for all the varieties (1.59 to 2.98 t.ha^-1^) was much higher compared to the total grain yield at Yusipang (0.61 to 2.68 t.ha^-1^). In the particular case of 2016 year, the means of grain yield of the same 10 varieties were also evaluated in six different locations namely Yusipang, Haa, Dawakha, Khangma, Metsham and Trashiyangtse Bhutan representing different agroecologies (Table 6). These grain yields varied from 1.22 to 2.57 t.ha^-1^ [4] but no significant difference were observed among the varieties (at p ≤ 0.05) for all the sites. This result signifies that there is not any variety that can perform better than the others can across all the sites as related by Bazile, Pulvento *et al*. [41] for their worldwide evaluation of quinoa in nine countries.

**Table 6:** Maturity and mean yield from six location in Bhutan for 10 quinoa varieties during 2016.

Variety	Days to Maturity*	Grain Yield (t ha^-1^)
DoA-1-PMB-2015	112.00^a^	2.52
Ivory 123	133.50^ab^	1.22
Salcedo INIA	154.17^ab^	1.24
INIA 420 Negra Collana	164.83^bc^	1.33
INIA 415 Pasankalla	168.50^bc^	1.51
Hualhuas	174.00^bc^	2.39
INIA 427 Amarilla Saccaca	173.67^bc^	2.57
Amarilla Maragani	174.83^bc^	1.91
Huancayo	175.50^bc^	2.23
Blanca de Junin	187.67^c^	1.77
p	**<0.001**	**ns**
SE ±	**13.79**	

*means followed by same letters are not significantly different

adapted from Katwal et al. [4].

In Serbia, Jankovic *et al*. [42] reported significantly fluctuation of quinoa grain yield, which ranged from 0.52 to 0.97 t.ha^-1^, and which was due to soil nutrient contents and varying levels of precipitation at different crop growth stages. In the Mediterranean climatic conditions in Turkey, experimental grain yields of quinoa has been found to range from 1.50 to 6.30 t.ha^-1^ where as in the farmers field grain yields ranged from 0.50 to 3.50 t.ha^-1^ [43]. In the Middle East and North African (MENA) countries, it has been reported that average yield of different quinoa genotypes varied between 1.2 and 1.4 t.ha^−1^, while the maximum attainable yield is predicted to go up to 8–10 t.ha^−1^ under controlled conditions [41, 44].

**Fig 2:**
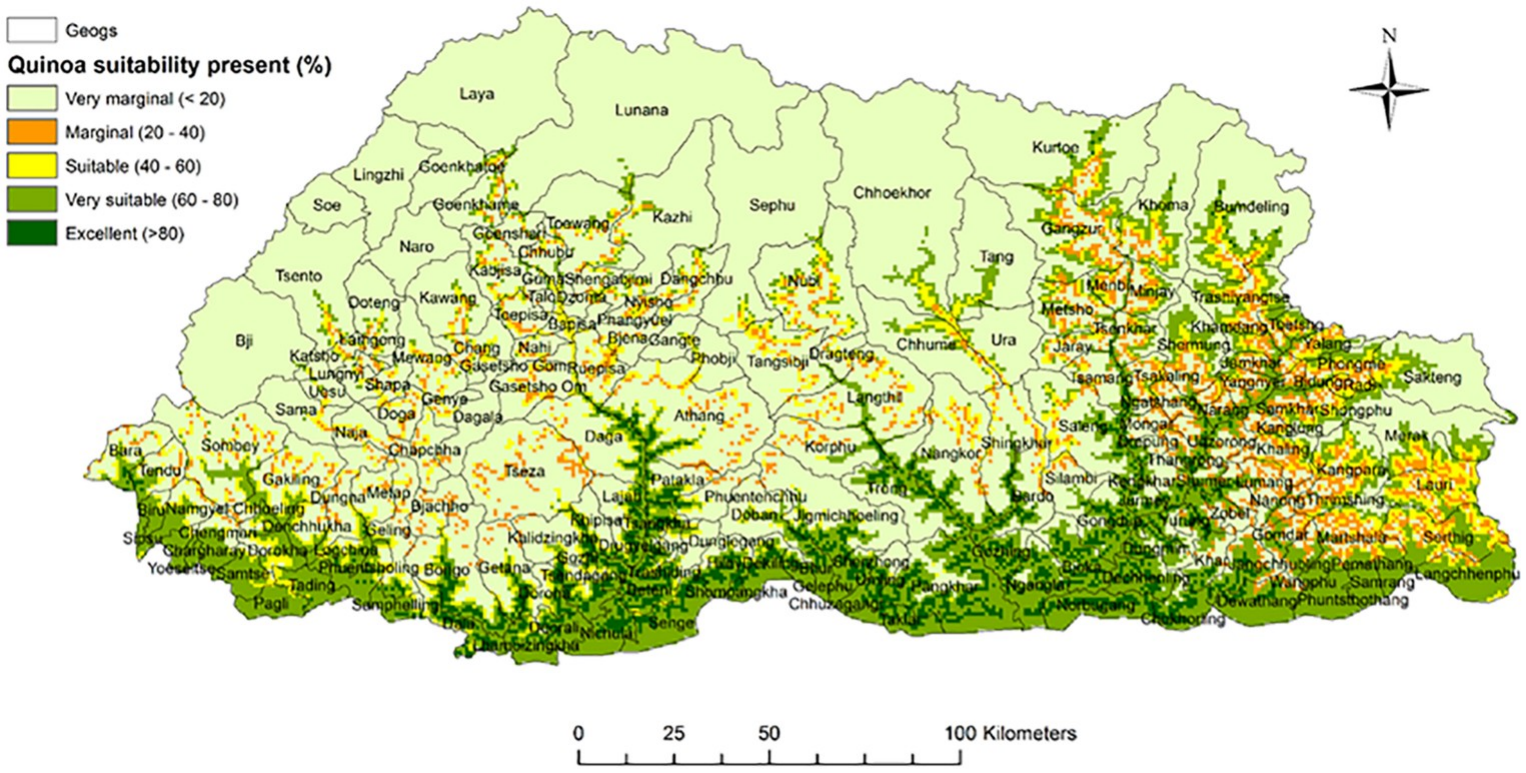
Quinoa suitability area under present conditions in Bhutan. Reprinted from [35] under a CC BY license, with permission from Louis Parker—CGIAR, original copyright 2017.

These trial results and other quinoa demonstrations indicate that quinoa can be successfully promoted under the Bhutanese mountain agriculture environments where temperature and total precipitation in the growing season varies from 4.4–19.1°C and 811 mm respectively in the cool temperate agroecology. In the humid sub-tropical agroecology temperature and total precipitation in the growing season varied from 9.4–32.4°C and 215 mm respectively. This result corroborates the assumptions of Bhargava *et al*. [15] in India that quinoa cultivation has very high potential to be expanded in the whole Himalayan region. Bhutanese farmers practice three distinct cropping systems, which are rice, maize and potato based systems [21], and where quinoa could be easily added for a sustainable crop diversification. Results from quinoa trials and demonstration conducted in Bhutan in 2015 and 2016/2017 seasons indicated that quinoa can adapt well and, could be grown as an alternative crop in both potato and maize based cropping systems [4]. A study undertaken by Parker *et al*. [35] on the challenges and opportunities of climate change for the Bhutanese agriculture sector has also identified quinoa as a potential commodity that is suitable for diversification and expansion of crops in the Bhutanese mountain agriculture systems. This study has also developed a quinoa suitability map with areas identified for diversification with quinoa (Fig 2).

Quinoa is considered to possess resistance to many adverse abiotic stresses like drought, frost and salinity [29, 45, 46]. Saponins of seed coats confer to quinoa plants a natural biological protection against some pests and diseases, in the field and during the storage period, even if saponins need to be removed before human consumption [47]. At Yusipang, the initiation of frost starts as early as the second fortnight of October and continues until the first fortnight of April. The effect of frost damage to quinoa was observed at Yusipang, which represents the cool temperate agroecology when crop was sown in the first fortnight of March and in August. Some preliminary observation trials on the cultivation of quinoa as a second crop after potato during in the warm and cool temperate areas has indicated that for these areas sowing has to be done by mid of July to escape the frost damage at anthesis. In quinoa crop, the flowering period that commences from first anthesis to the end of flowering is most sensitive to environmental stresses [48]. Further, it has been established that the effect of frost on seed yield of Quinoa can be as high as 51 to 66% when affected at 12 leaf stage and anthesis respectively [29]. It has been reported that despite quinoa’s potential to overcome many abiotic stresses some of the production challenges include sowing under the right conditions to obtain good crop stand from a small seeded crop. After a quick emergence, the management of weed is quite critical in quinoa. The storage and preservation of seed requires special attention because the embryo, with its external position has a short lifespan [8].

To fast track and rapidly aggressively promote quinoa as a climate resilient crop for food and nutritional security, four varieties have been released for cultivation under different cropping systems and agroecologies of Bhutan (Table 7). Of the four varieties, two are early maturity and two are medium to late in maturity.

**Table 7:** Quinoa varieties released for Bhutanese cropping systems.

Bhutanese name	Original name	Origin	Mean plant height (cm)	Maturity (days)	Grain color	Mean yield (t.ha^-1^)
Ashi Heychum- AM	Amarilla Marangani	Peru	188	173	Yellow	1.88
Ashi Heychum- AS	Amarilla Saccaca	Peru	165	170	Yellow	2.25
Ashi Heychum- 123	Ivory 123	India	122	150	Brownish	2.25
Ashi Heychum- TW	DoA-1-PMB-2015	Unknown	120	140	Brownish	1.88

adapted from Katwal [49]

As quinoa has been recently introduced in Bhutan, local farmers do not have the knowledge on its utilization. Researchers and extension staff are promoting the consumption of this nutritious crop by demonstrating the preparation of local Bhutanese dishes using milled quinoa grains. During farmers’ field days and local festivals, *Quinoa Thuep* (locally flavored soup), *Quinoa Dresi* (quinoa mixed with rice, cooked in butter and dressed with cashew nut), *Quinoa Kheer* (quinoa mixed with rice, cooked in milk and flavored with sugar) and *Quinoa Salad* were tried. These local Bhutanese dishes were prepared and served to encourage farmers and consumers to use quinoa for consumption. These steps are necessary to promote quinoa as a high nutritious crop for enhancing the food and nutritional security of the Bhutanese population. The overall feedback on the taste of quinoa dishes from the farmers and consumers is very positive and encouraging. As farmers and consumers have appreciated these initiatives, they are increasingly using quinoa for consumption by mixing it with their local staples like rice and maize.

Recognizing the high nutritional value of quinoa, the national government has also included quinoa in the school feed programme. The adaptation and rapid promotion of quinoa for food and nutritional security in the subsistence mountain agriculture systems of Bhutan in a small way contributes towards the global goal of “Great Food Transformation” which aims to promote healthy diet from a sustainable production system. One of the proposed strategies for the great food transformation is to reorient agricultural priorities towards producing healthy food rather than continuous focus on producing high quantity [50]. Further, *Chenopodium quinoa* is known to possess saponin, a naturally occurring plant *glycosides*, that has a very high medical application due to its anti-cancer properties [51]. These attributes of quinoa broadens the scope and prospects for the subsistence Bhutanese farmers to cultivate quinoa for food as well as for diversified markets.

## Conclusions

Quinoa, a new crop in Bhutan, has been successfully adapted and acclimatized in just over three years since its first introduction in 2015 to the unique mountain agriculture of this country in the Himalayas. Due to the versatile capacity of the crop to adapt to different growing conditions, quinoa has successfully adapted under the challenging mountain farming environments where different abiotic stresses, like varying precipitation patterns, drought, high temperature, frost and cold temperature, limit farmers’ choices of food crops and ability to ecological intensification of the cropping systems. The cultivation has been successfully demonstrated to the farmers across the country through on-farm demonstration and four varieties have been recommended with an indicative sowing time for the different agroecological zones and cropping systems. In the higher elevations above 1800 masl, spring sowing has been found most feasible. In these areas, a winter crop of quinoa after the harvest of main crops like potato and wheat has been partially successful but more research needs to be done for identifying genotypes and critical seeding time so that quinoa can escape frosts before anthesis. In the lower elevations below 1800 masl, sowing in autumn in the rainfed dryland, where quinoa is cultivated as a rainfed crop after the harvest of potato and maize, produced the best results. A winter crop after rice harvest in the terraced paddy fields is currently being evaluated with different genotypes. In this system, there is a need to identify suitable varieties with a growing period of less than 150 days, right sowing time and crop appropriate management practices.

From a new crop in 2015, quinoa has already spread in all 20 Dzongkhags of the country and across all agroecological zones with an estimated area of about 500 acres in 2018 (200 ha). Quinoa is now aggressively being promoted as a climate resilient and a nutrient dense crop across the country in all the agroecological zones to enhance food and nutritional security, and as a potential niche organic crop for the regional export market (China & India).

Quinoa is a new crop for the Bhutanese farmers and the overall understanding and management practices of this crop for their local mountain farming environments is still too poor for them. As the crop has not been previously evaluated in depth in this Himalayan region, the technical information available on quinoa is also scanty, and the basic information needed to judge agricultural activities to be done is very often partial or absent. Despite a high priority accorded to the promotion of quinoa cultivation, an all-out approach of the agriculture research and extension, to rapidly upscale the cultivation of quinoa at national level, is faced with numerous challenges. Among them, we can note limited access to quinoa germplasm [5, 52, 53], absence of best crop management practices under subsistence mountain farming systems, lack of knowledge on pest and diseases, harvesting and processing. Research and extension services of the Department of Agriculture have to face and address these challenges for consolidating the quinoa value chain. There is a need for package of practices for quinoa crop when grown under different agroecological conditions, organic production and marginal environments.

The Department of Agriculture has initiated quinoa research and development in all 20 Dzongkhags to address the agronomic and seed related issues. Developing and implementing a long-term strategy and a program for managing quinoa cultivation and consumption on the principles of sustainable development imply to consider the whole agrifood system with a territorial approach. Being a new crop for Bhutanese people, the technical knowledge and awareness about quinoa’s nutritional benefits is very limited. Now, quinoa has been essentially introduced to enhance the food and nutritional security of the Bhutanese population by promoting it as nutritious health food, which requires its inclusion in the traditional cuisines through adequate research and demonstration efforts. In parallel with resolving agronomic issues [49], the absence of marketing channels to sell farmers’ small household surpluses, which is absent today, must be resolved.

## Supporting information

S1 Table Data Limithang. Measurements made in 2016 and 2017.
(PDF)

S2 Table Data Yusipang. Measurements made in 2016 and 2017.
(PDF)
