# Supplementary material for: First adaptation of quinoa in the Bhutanese mountain agriculture systems
Source: PLoS One. 2020 Jan 16;15(1):e0219804. doi: 10.1371/journal.pone.0219804 (PMC6964828; doi:10.1371/journal.pone.0219804)
Supplement: S1 Table — Measurements made in 2016 and 2017. (PDF) [file pone.0219804.s001.pdf]

No	Location	Year	Rep	Variety	Days to Maturity	Mean PTHT cm	Yield Kg/acre	Yield T/ha
1	Limithang	2016	1	Salcedo INIA	104	148,00	1052,22	2,63
2	Limithang	2016	1	INIA 420 Negra Collana	109	152,20	607,05	1,52
3	Limithang	2016	1	Huancayo	104	138,00	1092,69	2,73
4	Limithang	2016	1	Blanca De Junin	109	165,00	890,34	2,23
5	Limithang	2016	1	Hualhaus	109	157,40	1133,16	2,83
6	Limithang	2016	1	Amarilla Marangani	109	139,00	930,81	2,33
7	Limithang	2016	1	INIA 427 Amarilla Sacaca	96	150,00	1092,69	2,73
8	Limithang	2016	1	INIA 415 Pasankalla	88	149,00	607,05	1,52
9	Limithang	2016	1	Ivory 123	95	141,00	1133,16	2,83
10	Limithang	2016	2	Salcedo INIA	104	134,00	1052,22	2,63
11	Limithang	2016	2	INIA 420 Negra Collana	109	125,00	607,05	1,52
12	Limithang	2016	2	Huancayo	104	135,00	1092,69	2,73
13	Limithang	2016	2	Blanca De Junin	109	177,40	890,34	2,23
14	Limithang	2016	2	Hualhaus	109	153,00	1133,16	2,83
15	Limithang	2016	2	Amarilla Marangani	109	145,00	930,81	2,33
16	Limithang	2016	2	INIA 427 Amarilla Sacaca	96	149,00	1092,69	2,73
17	Limithang	2016	2	INIA 415 Pasankalla	88	126,00	607,05	1,52
18	Limithang	2016	2	Ivory 123	95	128,00	1133,16	2,83
19	Limithang	2016	3	Salcedo INIA	104	129,00	930,81	2,33
20	Limithang	2016	3	INIA 420 Negra Collana	109	151,00	1052,22	2,63
21	Limithang	2016	3	Huancayo	104	136,00	1214,1	3,04
22	Limithang	2016	3	Blanca De Junin	109	151,00	930,81	2,33
23	Limithang	2016	3	Hualhaus	109	149,00	971,28	2,43
24	Limithang	2016	3	Amarilla Marangani	109	150,00	1173,63	2,93
25	Limithang	2016	3	INIA 427 Amarilla Sacaca	96	142,00	890,34	2,23
26	Limithang	2016	3	INIA 415 Pasankalla	88	138,00	1133,16	2,83
27	Limithang	2016	3	Ivory 123	95	146,00	1456,92	3,64
28	Limithang	2017	1	Salcedo INIA	104	78,00	169,97	0,42
29	Limithang	2017	1	INIA 420 Negra Collana	104	99,00	518,02	1,30
30	Limithang	2017	1	Huancayo	124	115,00	534,20	1,34
31	Limithang	2017	1	Blanca De Junin	128	114,00	295,43	0,74
32	Limithang	2017	1	Hualhaus	128	132,00	1088,64	2,72
33	Limithang	2017	1	Amarilla Marangani	119	124,00	534,20	1,34
34	Limithang	2017	1	INIA 427 Amarilla Sacaca	107	129,00	692,04	1,73
35	Limithang	2017	1	INIA 415 Pasankalla	96	73,00	404,70	1,01
36	Limithang	2017	1	Ivory 123	104	112,00	839,75	2,10
37	Limithang	2017	2	Salcedo INIA	104	96,00	477,55	1,19
38	Limithang	2017	2	INIA 420 Negra Collana	104	86,00	647,52	1,62
39	Limithang	2017	2	Huancayo	124	141,00	963,19	2,41
40	Limithang	2017	2	Blanca De Junin	128	133,00	756,79	1,89
41	Limithang	2017	2	Hualhaus	128	131,00	938,90	2,35
42	Limithang	2017	2	Amarilla Marangani	119	134,00	821,54	2,05
43	Limithang	2017	2	INIA 427 Amarilla Sacaca	107	137,00	777,02	1,94
44	Limithang	2017	2	INIA 415 Pasankalla	96	73,00	647,52	1,62

45	Limithang	2017	2	Ivory 123	104	118,00	1100,78	2,75
46	Limithang	2017	3	Salcedo INIA	104	109,00	603,00	1,51
47	Limithang	2017	3	INIA 420 Negra Collana	104	98,00	623,24	1,56
48	Limithang	2017	3	Huancayo	124	139,00	1133,16	2,83
49	Limithang	2017	3	Blanca De Junin	128	133,00	914,62	2,29
50	Limithang	2017	3	Hualhaus	128	139,00	582,77	1,46
51	Limithang	2017	3	Amarilla Marangani	119	150,00	1331,46	3,33
52	Limithang	2017	3	INIA 427 Amarilla Sacaca	107	131,00	1141,25	2,85
53	Limithang	2017	3	INIA 415 Pasankalla	96	77,00	404,70	1,01
54	Limithang	2017	3	Ivory 123	104	114,00	1481,20	3,70
