# Supplementary material for: First adaptation of quinoa in the Bhutanese mountain agriculture systems
Source: PLoS One. 2020 Jan 16;15(1):e0219804. doi: 10.1371/journal.pone.0219804 (PMC6964828; doi:10.1371/journal.pone.0219804)
Supplement: S2 Table — Measurements made in 2016 and 2017. (PDF) [file pone.0219804.s002.pdf]

No	Year	Location	Rep	Variety	Days to Maturity	Mean PTHT cm	Yield kg/acre	Yield t/ha
1	2016	Yusipang	1	Salcedo INIA	175	120,60	480	1,20
2	2016	Yusipang	1	INIA 420 Negra Collana	175	119,20	360	0,90
3	2016	Yusipang	1	Huancayo	185	167,00	400	1,00
4	2016	Yusipang	1	Blanca De Junin	185	176,20	240	0,60
5	2016	Yusipang	1	Hualhaus	185	165,00	640	1,60
6	2016	Yusipang	1	Amarilla Marangani	185	167,00	440	1,10
7	2016	Yusipang	1	INIA 427 Amarilla Sacaca	185	150,00	400	1,00
8	2016	Yusipang	1	INIA 415 Pasankalla	185	119,20	840	2,10
9	2016	Yusipang	1	Ivory 123	150	97,00	1800	4,50
9.1	2016	Yusipang	1	DoA-1- PMB -2015	102	107	642	1,44
10	2016	Yusipang	2	Salcedo INIA	175	125,80	640	1,60
11	2016	Yusipang	2	INIA 420 Negra Collana	175	106,80	680	1,70
12	2016	Yusipang	2	Huancayo	185	174,20	600	1,50
13	2016	Yusipang	2	Blanca De Junin	185	150,60	480	1,20
14	2016	Yusipang	2	Hualhaus	185	202,00	1120	2,80
15	2016	Yusipang	2	Amarilla Marangani	185	144,80	280	0,70
16	2016	Yusipang	2	INIA 427 Amarilla Sacaca	185	184,00	1360	3,40
17	2016	Yusipang	2	INIA 415 Pasankalla	185	129,00	160	0,40
18	2016	Yusipang	2	Ivory 123	150	104,00	1000	2,50
18.1	2016	Yusipang	2	DoA-1- PMB -2015	98	103	650	1,38
19	2016	Yusipang	3	Salcedo INIA	175	107,20	160	0,40
20	2016	Yusipang	3	INIA 420 Negra Collana	175	97,00	280	0,70
21	2016	Yusipang	3	Huancayo	185	152,80	240	0,60
22	2016	Yusipang	3	Blanca De Junin	185	133,40	80	0,20
23	2016	Yusipang	3	Hualhaus	185	159,00	600	1,50
24	2016	Yusipang	3	Amarilla Marangani	185	181,00	1120	2,80
25	2016	Yusipang	3	INIA 427 Amarilla Sacaca	185	213,80	1080	2,70
26	2016	Yusipang	3	INIA 415 Pasankalla	185	172,00	1120	2,80
27	2016	Yusipang	3	Ivory 123	150	166,00	1000	2,50
27.1	2016	Yusipang	3	DoA-1- PMB -2015	103	105,5	660	1,53
28	2017	Yusipang	1	Salcedo INIA	115	181,00	400	1,00
28	2017	Yusipang	1	INIA 420 Negra Collana	181	105,00	360	0,90
29	2017	Yusipang	1	Huancayo	208	110,00	200	0,50
30	2017	Yusipang	1	Blanca De Junin	208	125,00	240	0,60
31	2017	Yusipang	1	Hualhaus	208	115,00	320	0,80
32	2017	Yusipang	1	Amarilla Marangani	208	130,00	600	1,50
33	2017	Yusipang	1	INIA 427 Amarilla Sacaca	208	130,00	400	1,00
34	2017	Yusipang	1	INIA 415 Pasankalla	181	100,00	160	0,40
35	2017	Yusipang	1	Ivory 123	106	100,00	840	2,10
36	2017	Yusipang	1	DoA-1- PMB -2015	99	105,00	640	1,60
37	2017	Yusipang	2	Salcedo INIA	115	110,00	160	0,40
38	2017	Yusipang	2	INIA 420 Negra Collana	181	110,00	200	0,50
39	2017	Yusipang	2	Huancayo	208	115,00	240	0,60
40	2017	Yusipang	2	Blanca De Junin	208	120,00	200	0,50
41	2017	Yusipang	2	Hualhaus	208	112,00	480	1,20
42	2017	Yusipang	2	Amarilla Marangani	208	130,00	280	0,70

43	2017	Yusipang	2	INIA 427 Amarilla Sacaca	208	125,00	400	1,00
44	2017	Yusipang	2	INIA 415 Pasankalla	181	105,00	240	0,60
45	2017	Yusipang	2	Ivory 123	106	110,00	920	2,30
46	2017	Yusipang	2	DoA-1- PMB -2015	99	100,00	680	1,70
47	2017	Yusipang	3	Salcedo INIA	115	145,5	280	0,70
48	2017	Yusipang	3	INIA 420 Negra Collana	181	107,5	280	0,70
49	2017	Yusipang	3	Huancayo	208	112,5	220	0,55
50	2017	Yusipang	3	Blanca De Junin	208	122,5	220	0,55
51	2017	Yusipang	3	Hualhaus	208	113,5	400	1,00
52	2017	Yusipang	3	Amarilla Marangani	208	130	440	1,10
53	2017	Yusipang	3	INIA 427 Amarilla Sacaca	208	127,5	400	1,00
54	2017	Yusipang	3	INIA 415 Pasankalla	181	102,5	200	0,50
55	2017	Yusipang	3	Ivory 123	106	105	880	2,20
56	2017	Yusipang	3	DoA-1- PMB -2015	99	102,5	660	1,65
